# Hyaluronic acid and multiwalled carbon nanotubes as bioink additives for cartilage tissue engineering

**DOI:** 10.1038/s41598-023-27901-z

**Published:** 2023-01-12

**Authors:** Tomasz Szymański, Julia Anna Semba, Adam Aron Mieloch, Piotr Cywoniuk, Marcelina Kempa, Jakub Dalibor Rybka

**Affiliations:** 1 Center for Advanced Technology, Adam Mickiewicz University, Poznan, Poland; 2 Faculty of Chemistry, Adam Mickiewicz University, Poznan, Poland; 3 Faculty of Biology, Adam Mickiewicz University, Poznan, Poland

**Keywords:** Nanobiotechnology, Regenerative medicine, Tissue engineering, Nanoscale materials

## Abstract

Articular cartilage and meniscus injuries are prevalent disorders with insufficient regeneration responses offered by available treatment methods. In this regard, 3D bioprinting has emerged as one of the most promising new technologies, offering novel treatment options. Additionally, the latest achievements from the fields of biomaterials and tissue engineering research identified constituents facilitating the creation of biocompatible scaffolds. In this study, we looked closer at hyaluronic acid and multi-walled carbon nanotubes as bioink additives. Firstly, we assessed the minimal concentrations that stimulate cell viability, and decrease reactive oxygen species and apoptosis levels in 2D cell cultures of normal human knee articular chondrocytes (NHAC) and human adipose-derived mesenchymal stem cells (hMSC-AT). In this regard, 0.25 mg/ml of hyaluronic acid and 0.0625 mg/ml of carbon nanotubes were selected as the most optimal concentrations. In addition, we investigated the protective influence of 2-phospho-L-ascorbic acid in samples with carbon nanotubes. Tests conducted on 3D bioprinted constructs revealed that only a combination of components positively impacted cell viability throughout the whole experiment. Gene expression analysis of *COL1A1*, *COL6A1*, *HIF1A*, *COMP*, *RUNX2,* and *POU5F1* showed significant changes in the expression of all analyzed genes with a progressive overall loss of transcriptional activity in most of them.

## Introduction

Joint degeneration resulting from articular cartilage and meniscus defects is one of the most prevalent disorders of the musculoskeletal system. Cartilage’s low healing capacity and poor regeneration effects with available treatments motivate further research into new solutions^1^. A part of the effort is directed at new surgical techniques and more advanced biomaterials, facilitating the creation of biocompatible cartilage scaffolds^2^. Raising interest is observed in the field of biologically active materials, focused on the maintenance of cell viability and proper phenotype. Collagens in cartilage tissue form complex extracellular scaffolds to bear mechanical forces, maintain homeostasis and provide anchoring sites for chondrocytes, extracellular matrix (ECM) molecules, and growth factors^3^. For many years collagen was considered to be only a structural component of the cartilage matrix, but recently, its role in extracellular signaling, mainly via integrin receptors, was discovered^4^. Collagens regulate chondrocyte proliferation, metabolism, and differentiation; similarly to soluble signaling molecules. Additionally, they significantly suppress chondrocyte hypertrophy, which is the pathological process in osteoarthritic cartilage, leading to cell senescence and death. Interestingly, carbon nanotubes (CNTs) share similar dimensions to collagen fibrils, rendering them a potential collagen biomimetic^5,6^. Hyaluronic acid (HA) is another major ECM component, performing a dual role as a structural and signaling molecule. It has exceptional water retention properties, forming a gel-like environment within the tissue, and providing elasticity for the whole structure^7^.

In this study, we investigated multi-walled carbon nanotubes (MWCNTs) and hyaluronic acid (HA) as bioink additives for the 3D bioprinting of cartilage constructs. 3D bioprinting is a tissue engineering technology, which allows for precise spatial deposition of cell-enriched biomaterials, and recreation of tissue-specific structures capable to restore, maintain or improve damaged tissue through 3D scaffolds^2^.

Due to their unique biological and mechanical properties, carbon nanotubes are the subject of research in cartilage regenerative medicine. The MWCNTs are concentrically rolled graphene layers forming a cylindrical structure. Surfaces of synthetic materials functionalized with CNTs stimulate chondrocyte growth and facilitate the maintenance of their native phenotype^8,9^. The incorporation of CNTs also improves the mechanical properties of constructs, rendering them useful in scaffold reinforcement^10^. Most studies emphasize concentration-dependent effects. The cytotoxicity of carbon nanotubes is frequently emphasized and observed through an increase in reactive oxygen species (ROS) production. To mitigate cellular damage elicited by high ROS levels, antioxidant compounds such as l-ascorbic can be utilized. Due to its additional role in collagen production, a more stable analog, 2-phospho- l-ascorbic acid, was used in our study^11–13^.

In the case of cartilage tissue engineering, ECM components are of special interest not only due to their mechanical properties, ensuring the physiological functioning of cartilage as a shock absorber, but also due to their biological properties. Hyaluronic acid (HA) is a glycosaminoglycan prevalent in abundance in ECM of articular cartilage^14^. It provides antioxidative, anti-inflammatory, and chondroprotective effects, which are beneficial for cartilage repair. The concentration and molecular weight of HA determines its biological activity. This component was previously utilized as an additive to bioprint the articular cartilage constructs with auspicious stimulation of cell viability and phenotype^15^. HA has also been chemically modified to improve its mechanical, and biological properties, or enable UV cross-linking^16,17^.

This study assesses the chondrogenic properties of multi-walled carbon nanotubes and hyaluronic acid as bioink additives. In the first step, the minimal stimulating concentration of these components was determined with the 2D culture of normal human knee articular chondrocytes (NHAC) and human adipose-derived mesenchymal stem cells (hMSC-AT). Then, cell-containing 3D constructs were created using extrusion-based 3D bioprinting and formulated bioink. The bioink composition was based on alginate, gelatin, and carboxymethylated cellulose nanocrystals (CCNC), supplemented with MWCNTs or HA. Subsequently, the viability and gene expression of chondrogenesis markers were evaluated.

## Methods

### MWCNTs functionalization

MWCNTs were purchased and characterized as described in our previous work^18^. MWCNTs with diameters of 15–30 nm, lengths of 15–20 μm, and purity up to 95% produced by chemical vapor deposition (CVD) were functionalized by oxidation according to the following method. 30 mg of MWCNTs were sonicated at 70 °C in a mixture of concentrated sulfuric (H_2_SO_4_) and nitric (HNO_3_) acids in a ratio of 3:1. Then, the mixture was neutralized with 300 ml of 3 M sodium hydroxide (NaOH). Purification of the oxidized carbon nanotubes was carried out in cycles of centrifugation and resuspension in miliQ water at 9000×*g* at 20 °C for: (a) 15 min, (b) 30 min, (c) 40 min, and then 12,000×*g* at 4 °C for 40 min. The resulting carbon nanotube solution was dried using a vacuum evaporator. The MWCNTs were suspended in a phosphate buffer (PBS). The mass of nanotubes in a given volume of the solution was determined by the thermogravimetric method to calculate the concentration of the functionalized MWCNTs (which was 2.02 mg/ml).

### Cell culture

Normal Human Articular Chondrocytes (NHAC, LONZA Catalog #: CC-2550) were cultured in CGM™ Chondrocyte Growth Medium (LONZA), while human adipose tissue-derived mesenchymal stem cells (hMSC-AT, PromoCell) were cultured in supplemented Mesenchymal Stem Cells Growth Medium 2 (PromoCell); both in a humidified 5% CO_2_ atmosphere, at 37 °C in tissue culture flasks (Falcon®). The culture medium was changed every three days and cells were passaged with TrypLE (Gibco) at 80–85% confluency. Additionally, hMSC-AT were cultured in Mesenchymal Stem Cell Chondrogenic Differentiation Medium (PromoCell) as a reference for gene expression analysis.

### Determination of the cell viability, reactive oxygen species, and apoptosis levels in 2D cell cultures stimulated with MWCNTs and 2-phospho-l-ascorbic acid

For all tests, NHAC and hMSC-AT were seeded in a clear bottom 96-well plate (Corning) at a density of 1000 cells/well. After 24 h, medium with 0.015, 0.03, 0.0625, and 0.125 mg/ml MWCNTs was added to each well; the total volume was 100 μl medium/well. To investigate the antioxidant effect of vitamin C, the same replicates were performed with the addition of 50 μg/ml.

2-phospho-L-ascorbic acid in a cell culture medium. This salt was used instead of regular ascorbic acid, due to its increased stability in water solutions^19^. The viability was assessed with the CellTiter-Glo® Luminescent Cell Viability Assay (Promega) as described above.

The level of H_2_O_2_ and reactive oxygen species (ROS) were determined according to the manufacturer protocol of the ROS-Glo™ H_2_O_2_ Assay (Promega). Briefly, 24 h after seeding, 20 μl of H_2_O_2_ substrate solution was added to each well. The samples were incubated for 2 h at 37 °C with 5% CO_2_. Subsequently, 100 μl of ROS-Glo™ Detection Solution was added to each well, and samples were incubated for 30 min at room temperature. The luminescent signal was read with a microplate reader (Infinite® 200 PRO, TECAN). ROS-Glo™ H_2_O_2_ Assay results were correlated with the CellTiter-Glo® Luminescent Cell Viability Assay. In an analogical way, Caspase-Glo® 3/7 Assay Systems (Promega) were conducted to investigate apoptosis via caspase activity. All samples were conducted in triplicate. The luminescence values were normalized to respective control samples (100%). The statistical significance was determined by a two-tailed Student’s t-test (n = 3; additive vs control: *P < 0.05; **P < 0.01 and ***P < 0.001).

### Determination of cell viability in 2D cultures supplemented with HA

For all experiments, NHAC, and hMSC-AT cells were seeded in a clear bottom 96-well plate (Corning) at a density of 1000 cells/well. After 24 h, medium with 0.125, 0.25, 0.5, or 1 mg/ml HA (Contipro) was added to each well; the total volume was 100 μl medium/well. Cells were cultured in the supplemented medium at 37 °C with 5% CO_2_. The experiment was performed according to the producer protocol of the CellTiter-Glo® Luminescent Cell Viability Assay (Promega). CGM™ medium and Mesenchymal Stem Cells Growth Medium 2 without HA were used as a control. After 24 h, 48 h, and 72 h, 100 μl of CellTiter-Glo® Reagent was added to each well. The samples were incubated for 30 min at room temperature. The luminescent signal was read with a microplate reader (Infinite® 200 PRO, TECAN). All samples were conducted in triplicate. The luminescence values were normalized to respective control samples (100%). The statistical significance was determined by a two-tailed Student’s t-test (n = 3; additive vs control: *P < 0.05; **P < 0.01 and ***P < 0.001).

### The preparation of bioink

The bioink was prepared as follows. Weighted and UV-sterilized sodium alginate (Sigma-Aldrich) was dissolved in a sterile 4.6% (w/v) D-mannitol (Sigma-Aldrich) solution. Subsequently, weighted and UV-sterilized porcine skin gelatin (Sigma-Aldrich), and CCNC (Cellulose Lab) were separately added and mixed with the alginate solution with two syringes connected with the female/female Luer-lock adapter. The materials were shaken each time at 37 °C for at least 30 min with HulaMixer™ Sample Mixer, followed by overnight mixing. The final concentrations of bioink components were 4.0% gelatin, 0.75% alginate, and 1.4% CCNC. Before adding cells, HA or/and MWCNTs were added and bioink was additionally mixed with two syringes connected with the female/female Luer-lock adapter. The prepared bioink was mixed with 8 × 10^6^ cells/ml of bioink in an analogical way. Only hMSC-AT were utilized for 3D bioprinting. Before bioprinting, the bioink with cells was placed in a cartridge and held at a 25 °C water bath to induce gelatin gelation.

### The rheological tests

The rheological evaluation was performed on Anton Paar 302 rheometer, equipped with 25 mm, smooth, parallel plates (PP25) with bioink without cells and before crosslinking. The gap between plates was set to 1 mm and—unless stated otherwise—measurements were conducted at 23 °C. Performed rheological measurements were temperature sweep test and rotation. Temperature sweep experiments were performed at a rate of 2 °C/min from 20 to 40 °C. In the rotation study, the shear rate range was set to 0.01–1000.00 s^−1^. A layer of silicone oil was spread over the verge surface of the sample to prevent water evaporation from bioink samples during rheological measurements. All rheological tests were performed in at least two repeats.

### 3D bioprinting

The BioX printer (Cellink) with temperature-control, pressure extrusion printhead was used, with printhead temperature set at 25 °C, and printbed temperature set at 10 °C.

After bioprinting, the constructs were crosslinked with sterile 200 mM CaCl_2_ (Sigma-Aldrich) dissolved in 4.6% (w/v) D-mannitol (Sigma-Aldrich) for 10 min at room temperature.

The constructs were cultured in Mesenchymal Stem Cell Growth Medium 2 (Promocell), and the Mesenchymal Stem Cell Chondrogenic Differentiation Medium (Promocell) in the case of cells used as differentiation control. The constructs were cultured in standard conditions (37 °C, 5% CO_2_) and the medium was changed every 3 days.

### SEM–EDX

The constructs bioprinted without cells were subjected to Scanning Electron Microscopy (SEM) with Energy Dispersive X-Ray Analysis (EDX). The analyzed scaffolds were with or without the addition of the MWCNTs. The morphology of samples was characterized by scanning electron microscope Quanta FEG 250 (FEI) in low vacuum conditions at the pressure of 70 Pa with an electron beam energy of 10 keV. EDS spectra were collected with an electron beam energy of 30 keV using an EDS Octane SDD detector (EDAX). Prior to analysis, the scaffolds were frozen at −80 °C for 2 h and then lyophilized (Christ, Alpha 1–2 LDplus lyophilizer) for 12 h, at the pressure of 1 mBar. Subsequently, the pressure was decreased to 0.18 mBar and the process was carried out for another 4 h.

### The live/dead assay of cells encapsulated in bioprinted construct

After 24 h, 14 days, and 21 days, bioprinted constructs were analyzed with the LIVE/DEAD assay performed according to the manufacturer`s protocol (LIVE/DEAD® Viability/Cytotoxicity Kit, Invitrogen). Stained cells were visualized on a confocal microscope (IX83, Olympus). Scans for viability counting were taken from the lateral part of three different constructs at each time point. From each of these scans, two middle slices were chosen for live and dead cell counting. Obtained images were analyzed with Fiji software using ITCN functionality. The viability was calculated as a % of live cells. The statistical significance was determined by two-tailed Student’s t-test (n = 3; additive vs control: P^a^ < 0.05; P^b^ < 0.01 and P^c^ < 0.001; timepoint vs timepoint (within the particular additive group): *P < 0.05; **P < 0.01 and ***P < 0.001).

### Analysis of gene expression of cell-laden constructs

Three constructs from each time point were dissolved in 100 mM sodium citrate containing 0.08 U/μl of Proteinase K and 1.0 U/μl of RNAse Inhibitor (A&A Biotechnology) with shaking at 37 °C for 5 min and followed by the RNA isolation with TriReagent (Sigma-Aldrich) and chloroform/phenol extraction. Isolated total RNA concentration was measured with the Qubit 4 fluorometer (Invitrogen) and reversely transcribed with the High-Capacity cDNA Reverse Transcription Kit (Thermo Scientific). Gene expression was analyzed from 7.5 ng of cDNA per sample with real-time PCR using Maxima SYBR Green/ROX qPCR Master Mix (Thermo Scientific) on QuantStudio 7 Flex (Applied Biosystems). The qPCR data were statistically analyzed with GraphPad Prism software. Relative expression was calculated with ddCt and referred to *RPS29* gene expression. The variations in gene expression were determined by two-tailed Student’s t-test (n ≥ 2); P-values were considered significant as follows: additive vs control: P^a^ < 0.05; P^b^ < 0.01 and P^c^ < 0.001; timepoint vs timepoint (within the particular additive group): *P < 0.05; **P < 0.01 and ***P < 0.001. Sequences of primers used are listed in Supp. Tab. 1.

## Results

### Analysis of viability, ROS, and caspase 3/7 generation in 2D NHAC and hMSC-AT cell cultures

**Figure 1:**
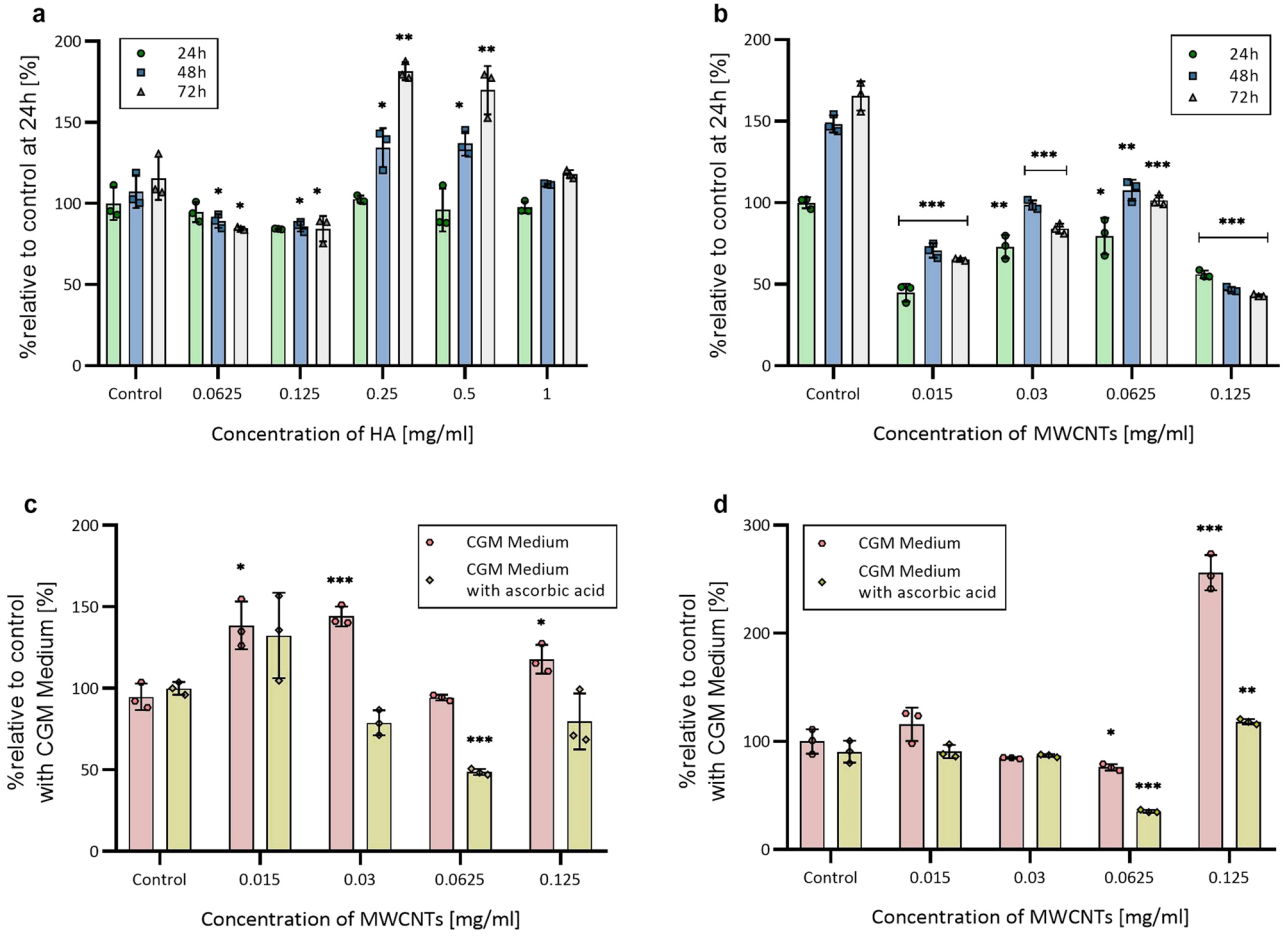
Relative cell viability, ROS levels and caspase 3/7 activity in 2D NHACs culture. (**a**) Relative viability of NHACs after exposure to different concentrations of HA. (**b**) Relative viability of NHACs after exposure to different concentrations of MWCNTs. (**c**) Relative ROS generation after 24 h exposure to MWCNTs. (**d**) Relative Caspase 3/7 activity after 24 h incubation with MWCNTs. The data are presented as the mean ± SD. The statistical significance was determined by two-tailed Student’s t-test (n = 3; additive vs control: *P < 0.05; **P < 0.01 and ***P < 0.001).

**Figure 2:**
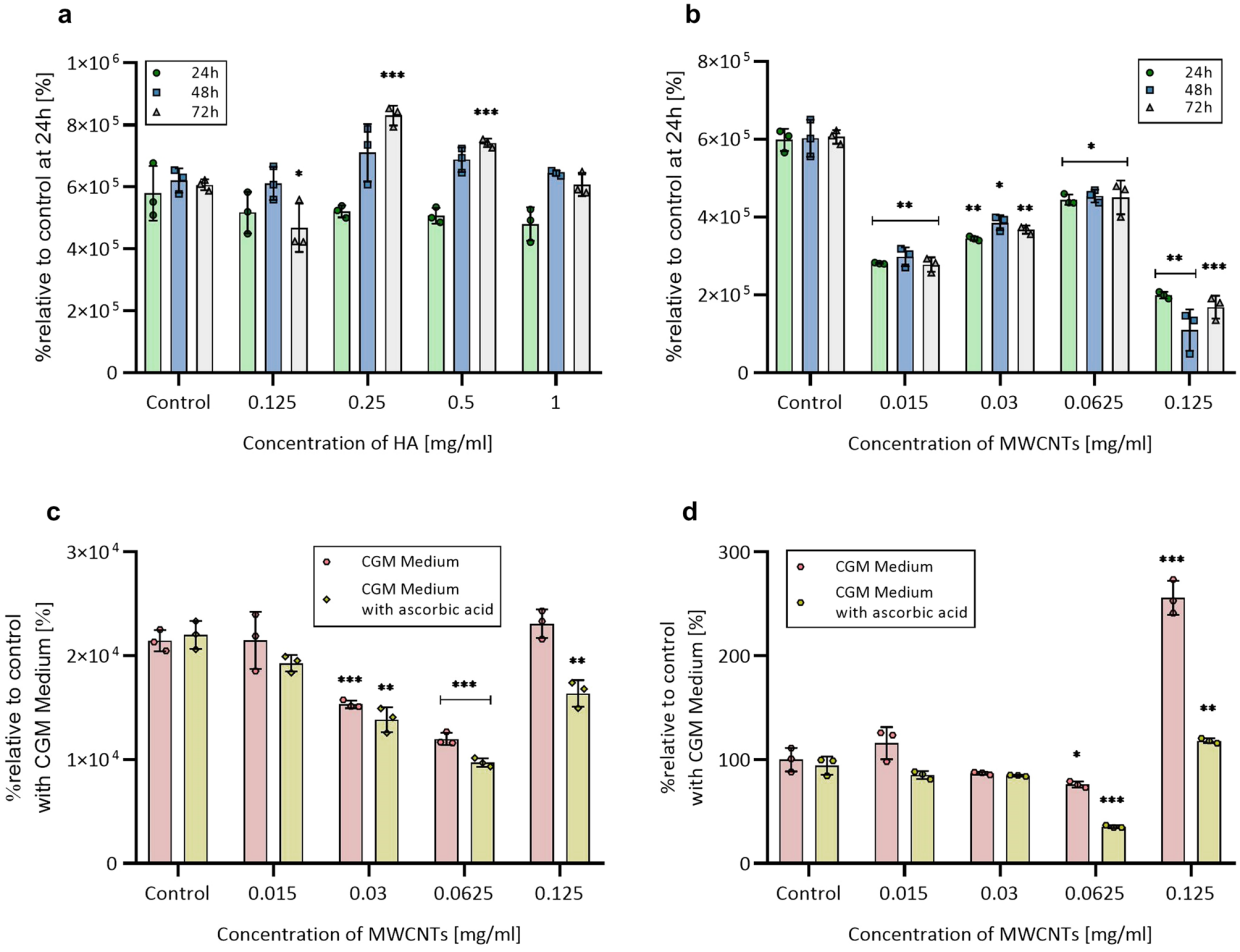
Relative cell viability, ROS levels, and caspase 3/7 activity in 2D hMSC-AT culture. (**a**) Relativeiability of hMSC-AT after exposure to different concentrations of HA. (**b**) Relative viability of hMSC-AT after exposure to different concentrations of MWCNTs. (**c**) Relative ROS generation after 24 h exposure to MWCNTs. (**d**) Relative caspase 3/7 activity after 24 h incubation with MWCNTs. The data are presented as the mean ± SD. The statistical significance was determined by two-tailed Student’s t-test (n = 3; additive vs control: *P < 0.05; **P < 0.01 and ***P < 0.001).

In NHAC 2D culture, 1 mg/ml of HA addition had no significant effect on cell proliferation (Fig. 1a). The culture exposed to 0.25 mg/ml of HA showed negligible changes in viability after 24 h. However, increased proliferation was observed after 72 h of culture. Similar results were obtained for hMSC-AT (Fig. 2a). The highest increase in proliferation was observed at the HA concentration of 0.25 mg/ml. A decrease in cell viability of both NHAC and hMSC-AT cultures was observed for all MWCNTs concentrations. The highest decrease of about 50% in viability was observed for 0.125 mg/ml. Interestingly, in the culture with the lower range of MWCNTs concentrations (0.015 mg/mL and 0.03 mg/ml), cell viability was diminished in comparison to a higher concentration of 0.0625 mg/ml at all time points (Figs. 1b and 2b). Observed dependency was corroborated by analyses of ROS production and active caspases 3/7 (Figs. 1 c,d and 2c,d). The stable form of vitamin C (2-phospho-l-ascorbic acid) yields protective and antioxidative effects in the NHAC and hMSC-AT cell culture with CNTs. The strongest antioxidant effect of vitamin C is seen at the highest concentration of MWCNTs (0.125 mg/ml). Its supplementation causes a significant reduction in the production of reactive oxygen species and inhibits cell death compared to the medium without 2-phospho-l-ascorbic acid supplementation (Figs. 1 c,d and 2c,d).

### Analysis of bioink and 3D bioprinting

The addition of 0.25 mg/ml HA and 0.0625 mg/ml MWCNTs yielded the most beneficial effect in 2D tests, therefore these concentrations were selected for 3D bioprinting. The aim was to assess whether selected additives influence cell viability and induce chondrogenic differentiation in 3D culture. The hMSC-AT cell line at a concentration of 8 × 10^6^ cells/ml was used in the study due to a higher proliferation rate than NHAC.

**Figure 3:**
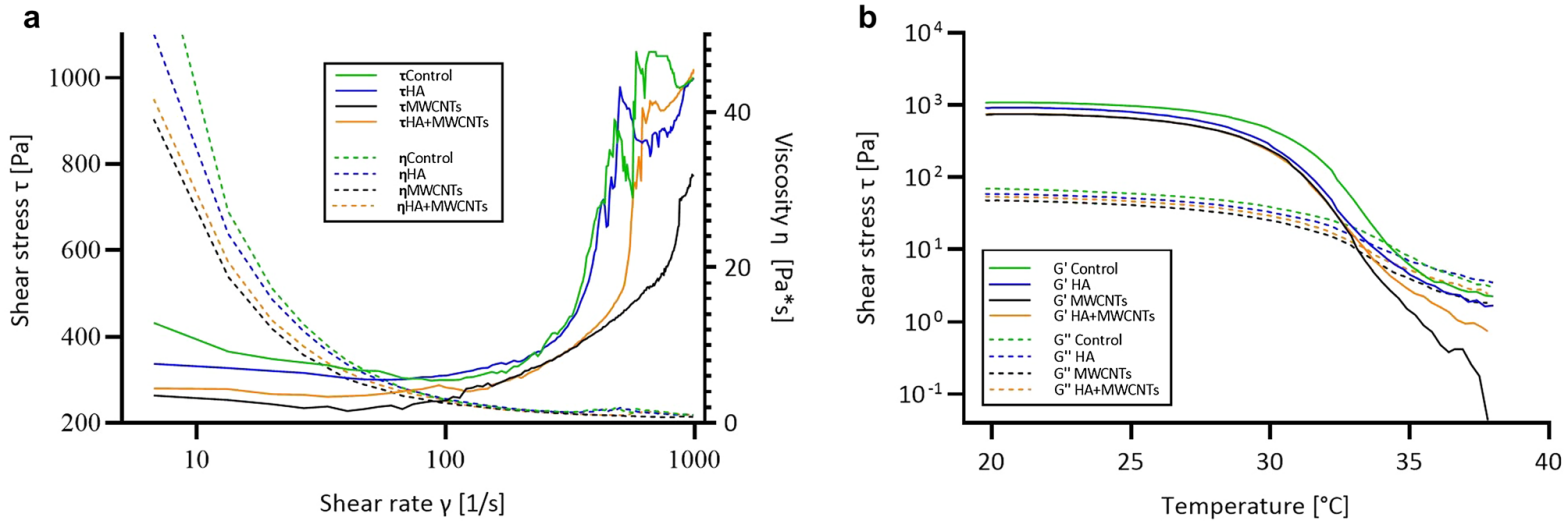
Rheological analysis of bioinks with MWCNTs or HA addition. Control is bioink without MWCNTs and HA. (**a**) Shear stress and viscosity as a function of shear rate. (**b**) The temperature-dependent functions of storage modulus G' and loss modulus G''.

The MWCNTs or HA addition showed negligible influence on the rheological properties of bioinks. All of them exhibit a shear-thinning behavior and have similar cross-over temperatures (G' = G''), which signifies good printability (Fig. 3). Table 1 presents parameters set for 3D bioprinting.

**Table 1:** 3D bioprinting parameters.

	No additives	Bioink with 0.25 mg/ml HA	Bioink with 0.0625 mg/ml MWCNTs	Bioink with 0.25 mg/ml HA and 0.0625 mg/ml MWCNTs
Needle	22 gauge (inner diameter = 410 µm)			
Pressure	60–68 kPa	65–70 kPa	55–60 kPa	65–70 kPa
Speed	14–15 mm/s	15–18 mm/s	14–15 mm/s	16–18 mm/s
Preflow	200 ms			
Postflow	0 ms			

SEM–EDX analysis showed insignificant changes in structure and elemental composition (Supp. Fig. 1). In the SEM images, regular pores can be observed, which match our bioprinting model. scaffolds also present highly fibrous structures.

### Cell viability in bioprinted scaffolds

**Figure 4:**
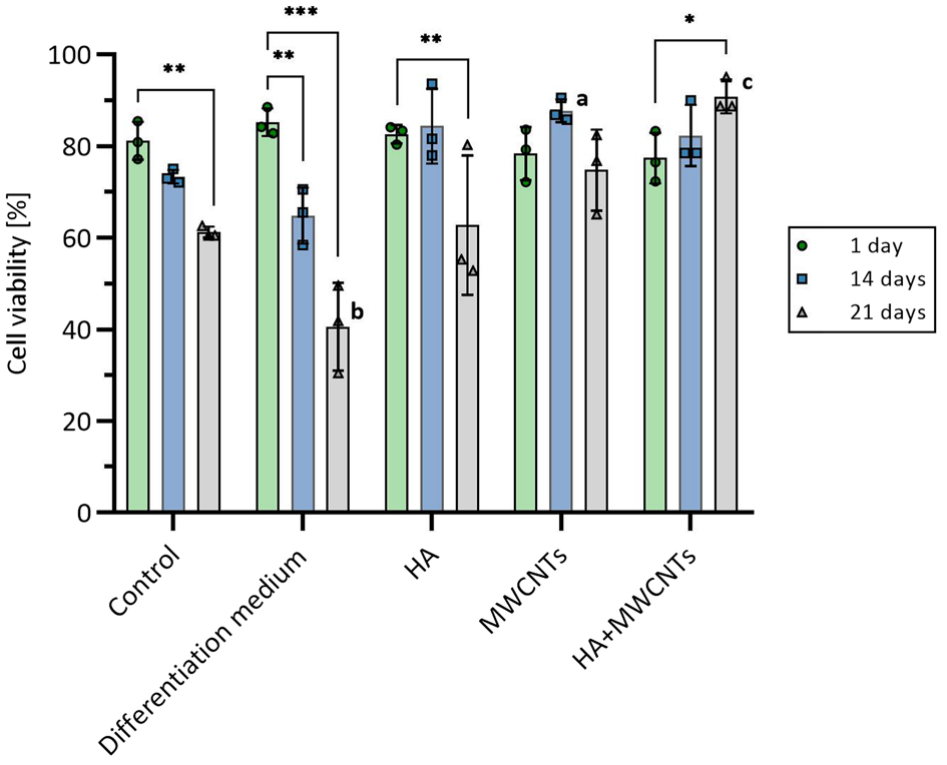
Viability of hMSC-AT cells in bioprinted scaffolds. Loss of viability over time can be observed in control media, supplemented with HA, and most profoundly, in commercial differentiation medium. Addition of the MWCNTs alone results in a protective or stimulating effect on cells, because the decrease in viability is not observed. Combined with HA, the viability has even increased over 21-day period. The data are presented as the mean ± SD. The statistical significance was determined by two-tailed Student’s t-test (n = 3; additive vs control: ^a^P < 0.05; ^b^P < 0.01 and ^c^P < 0.001; timepoint vs timepoint (within particular additive group): *P < 0.05; **P < 0.01 and ***P < 0.001).

Live/dead assay was performed in order to determine the hMSC-AT viability in the 3D scaffolds (Fig. 4). In the control medium, a constant decrease in total viability was observed. Interestingly, the biggest decline in viability was observed in the differentiation medium, despite the cells having the highest transcriptional activity (see paragraph 3.4.). A similar decline was observed in HA-enriched constructs. In the case of MWCNTs-containing constructs, the decline was not observed, which implies a stimulating or protective effect on the cells. HA and MWCNTs combined were the only compositions, showing a synergistic effect, which has a positive impact on cell viability throughout the whole experiment.

### HA and MWCNTs supplementation affect the expression of chondrogenic markers

**Figure 5:**
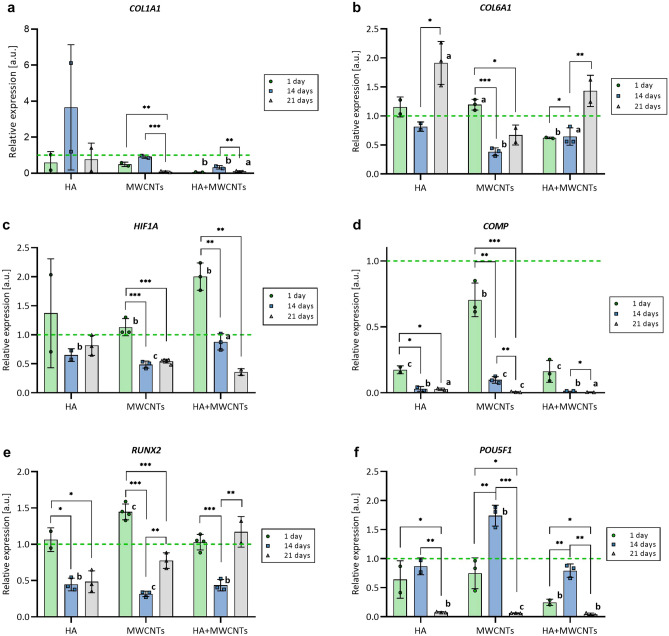
Bioink additives affect the expression of chondrogenic markers. Real-time analysis of COL1A1 (**a**), COL6A1 (**b**), HIF1A (**c**), COMP (**d**), RUNX2 (**e**) and POU5F1 (**f**) gene expression in hMSCs 3D-printed with bioink containing 0.25 mg/ml of hyaluronic acid (HA), 0.0625 mg/ml of multi-walled carbon nanotubes (MWCNTs) or mix of HA and MWCNTs (HA + MWCNTs) (0.25 mg/ml and 0.0625 mg/ml, respectively) 1 day (1d), 14 days (14d) and 21 days (21d) post-printing. The expression of each variant is normalized to the average expression in hMSCs 3D-printed with bioink without additives at a particular time point (green dashed line). The statistical significance was determined by two-tailed Student’s t-test (n ≥ 2; additive vs control (green dashed line): ^a^P < 0.05; ^b^P < 0.01 and ^c^P < 0.001; timepoint vs timepoint (within particular additive group): *P < 0.05; **P < 0.01 and ***P < 0.001).

To evaluate the chondrogenic potential of HA and functionalized MWCNTs, the gene expression analysis of genetic markers of chondrogenesis and stemness was performed (Fig. 5). Based on available data, the following genes were selected as chondrogenic markers: *COL1A1, COL6A1, COL10A1* and *COMP* (encoding collagen type I, VI, X, and Cartilage Oligomeric Matrix Protein, respectively—ECM components), *RUNX2*, *HIF1A,* and *SOX9*; (transcription factors)^3,20^. Additionally, to monitor the stemness of hMSCs we analyzed the expression of transcription factor Oct-4 (*POU5F1* gene)^21^. hMSC-AT-containing scaffolds of each bioink variant were maintained in culture for 1, 14, or 21 days. At each time point, expression of the hMSC-AT in bioink variants was normalized to hMSC-AT 3D bioprinted with bioink without additives (Fig. 5, green dashed lines). An additional group of control scaffolds was maintained in the chondrogenic medium for the same time intervals to define reference expression profiles in differentiated cells (DIFF) (Supp. Fig. 2). Gene expression profiles of cells from the same bioink variant between different time points were juxtaposed. A set of analyzed genes demonstrates expression fluctuations across subsequent time points (Fig. 5a–c,e,f) when compared to the control group (Fig. 5a–c,e,f, green dashed lines). The expression of two analyzed genes, *SOX9* and *COL10A1*, dropped below detectable levels on day 14 and 21, respectively, for all analyzed bioink variants including control scaffolds (data not shown), therefore, they were excluded from further analysis. Incubation for 21 days revealed an intense decrease of *COL1A1, HIF1A, COMP,* and *POU5F1* genes expression in MWCNTs and HA + MWCNTs bioink variants and *COMP* and *POU5F1* expression in HA variant (Fig. 5a,c,d,f). In parallel, expression analysis of the same genes in DIFF medium 21 days post-printing showed a rapid increase of *COL1A1* and *COMP* activity, and a significant decrease of *POU5F1* while expression of *HIF1A* remained unchanged (Supp. Fig. 2a,c,d,f). For HA and HA + MWCNTs variants, a significant increase in *COL6A1* expression was observed on day 21 (Fig. 5b) as well as for DIFF (Supp. Fig. 2b). The initial significant increase was also detected in *HIF1A* and *RUNX2* genes for MWCNTs and HA + MWCNTs (*HIF1A*) and MWCNTs (*RUNX2*) followed by a deep decrease on day 14 (Fig. 5c,e). For MWCNTs on day 21 expression of *RUNX2* was restored to the control level as well as for HA + MWCNTs, yet, without expression boost on day 1 (Fig. 5e). Expression of *RUNX2* in DIFF progressively elevated (Supp. Fig. 2e). Temporal transcriptional activation has also been observed for MWCNTs and HA + MWCNthe Ts in the *POU5F1* gene, where 14 days post-printing gene expression strongly increased to drop to a barely detectable level at day 21 (Fig. 5f) which reflects the expression profile of *POU5F1* in DIFF (Supp. Fig. 2f). Noteworthy, despite the same dynamics, *POU5F1* expression for HA + MWCNTs was much lower than for MWCNTs and did not reach the expression level observed in control scaffolds (Fig. 5f, 1 day, and 14 days).

## Discussion

Bioink development is an inextricable part of 3D bioprinting for tissue engineering. Structural materials, like alginate or cellulose, are responsible for construct integrity and proper mechanical features; whereas biologically active substances are added to maintain cell functionality or stimulate various biological effects. This work investigated the influence of HA and functionalized MWCNTs firstly in 2D culture and then as additives to bioink designed for cartilage regeneration.

The toxicity of carbon nanotubes is a frequently raised concern regarding its utilization in tissue engineering^22^. The addition of the MWCNTs in all tested concentrations decreased cell viability and resulted in an increased ROS production in 2D cultures (Figs. 1b and 2b). These results confirm our previous reports^18^. Interestingly, R OS production diminished with increasing concentrations of MWCNTs, but when the concentration exceeded 0.0625 mg/ml, a robust generation of ROS occurred. This phenomenon could be elucidated by ROS scavenging facilitated by the CNTs, as described earlier. Our previous research reveals the interference of CNTs with luminescence-based assays, yielding nonrepresentative results, falsely indicating the high toxicity of this nanomaterial. However, at low concentrations used in this study, this interference may be omitted. Additionally, combining the assay with the measurement of caspase activity provides a double check on the reliability of the assay.

Additionally, to mitigate oxidative stress elicited by the MWCNTs, a more stable analog of vitamin C was tested. It has been demonstrated that ascorbic acid promotes chondrogenic cell differentiation, and helps to maintain a chondrogenic phenotype, especially in pathological conditions^23^. Our study corroborates the beneficial effects of ascorbic acid on oxidative stress and the viability of cells.

Alginate-HA bioink has been previously utilized to bioprint articular cartilage constructs^15^. In the study, the authors demonstrated that HA addition increased chondrogenic gene expression; however, in a contrast to our experimental design, a thermoplastic polymer was used as a structural material. The addition of HA was shown to affect the viscosity of bioink and, consequently, printability^24^. The rheological analysis of our bioinks showed inconsiderable variation. This discrepancy could be explained by the relatively low concentration of HA in the bioink (0.25 mg/ml).

The lowest level of the MWCNTs cytotoxicity in 2D culture was observed at a concentration of 0.0625 mg/ml which was subsequently used for bioink formulation. Cells cultured in 3D bioprinted constructs showed higher tolerance to increasing concentrations of carbon nanotubes, compared to 2D culture^25^. In 3D culture, carbon nanotubes are embedded in a hydrogel matrix, which limits their ability to be absorbed by the cells, while in 2D cultures carbon nanotubes diffuse into the medium facilitating cellular uptake by endocytosis^26^. In our previous study, 0.01% of CNTs embedded in polycaprolactone scaffold increased chondrocyte adhesion and proliferation^8^.

Live/dead analysis showed that in the scaffold without additives (control and differentiation medium), as well as with HA, the viability of cells decreased over time. This is not the case in the scaffolds supplemented with MWCNTs. It may be attributed to the CNTs’ resemblance to collagen fibrils, forming a 3D intricate mesh-like structure (Supp. Fig. 1), which may have a stimulating effect on the cells. Partial degradation of the HA-supplemented scaffolds could be caused by an increase in water content due to the strong hydrophilicity of HA, leading to the loss of integrity and subsequent degradation. Scaffolds with both MWCNTs and HA showed the highest viability of cells. They were also more stable than HA-supplemented scaffolds. This observation can further corroborate the CNTs’ resemblance to collagens since collagen's main structural function is to provide tensile strength to the whole tissue.

In general, significant changes in expression of all analyzed genes with a progressive overall loss of transcriptional activity were observed (*COL1A1, HIF1A, COMP, POU5F1—*Fig. 5a,c,d,f, *COL10A1, SOX9—*data not shown). However, in DIFF samples the expression of *COL1A1* and *COMP* increased intensively while the *HIF1A* level remained stable (Supp. Fig. 2a,c,d). HIF1A protein is prone to oxygenation as a target of HIF hydroxylases and its level is elevated during hypoxia^27^. The initial increase of *HIF1A* expression observed on day 1 for all bioink variants may be due to culture format conversion from 2 to 3D which resulted in temporal hypoxia or hypoxia-like conditions, however, a stable level of *HIF1A* in DIFF does not support such hypothesis. On the other hand, the chondrogenic medium contains a variety of components that intensively stimulate the differentiation process (a detailed formulation of the medium was unavailable) some of which may exhibit antagonistic properties regarding *HIF1A* activation.

Since *HIF1A* positively regulates the expression of *SOX9*, it may explain the dramatic loss of its expression observed on days 14 and 21^28^. Collagen type I, VI, X, and COMP protein are constituents of cartilage ECM and their increased expression is observed at different stages of chondrogenic differentiation^3^. Although all bioink variants revealed an intense decrease in expression of *COL1A1* (except for HA, however, insignificant), *COL10A1,* and *COMP*, expression of *COL6A1* increased in HA and HA + MWCNTs. It also reflects a similar expression pattern in MWCNTs samples, yet, insignificant. It has been shown that the expression of *COL1A1* is prone to the presence of collagen type I-derived fragments^29,30^. As gelatin is one of the main components of the bioink formulation, it is plausible that short collagen fragments inhibit *COL1A1* transcription. A significant increase of *COL1A1* expression in DIFF might be, contrastingly, a result of the dominant stimulatory effect of the chondrogenic medium which ameliorates collagen fragment inhibitory properties. Little is known about the regulation of *COMP* expression. However, previous studies show that COMP binds to collagen type I which might point to mutual or synergistic regulation and explain the simultaneous decrease of *COMP* gene expression in all bioink variants, in contrast to DIFF where expression of both genes is dramatically increased^31^. Collagen type X is abundant in hypertrophic chondrocytes, therefore, intense loss of its expression in bioprinted hMSCs might be a hallmark of early-stage chondrogenic differentiation^32^. Although collagen type VI comprises up to 1% of total collagen in articular cartilage it plays important role in ECM organization and governing chondrocyte fate and, therefore, serves as an indicator of chondrogenesis^33–35^. The moderate increase observed in HA and HA + MWCNTs corresponds to changes in *COL6A1* observed in DIFF. Although the magnitude of change in *POU5F1* expression in MWCNTs and HA + MWCNTs is notably lower, its profile is similar to this observed in DIFF. Interestingly, while the *POU5F1* gene tends to deactivate in differentiating cells, its expression at day 14 in DIFF (as well as in CNTs and HA + MWCNTs) strongly elevates to almost complete deactivation on day 21. That might point to significant transcriptional rearrangement in hMSCs during incubation in a chondrogenic medium but in the presence of MWCNTs or a mix of HA and MWCNTs as well. Taken together, the addition of HA or MWCNTs, alone or in tandem, to the bioink provokes alterations in the expression of genes related to chondrogenic differentiation. Although the expression patterns are not identical for all selected genes and the magnitude of observed changes is considerably lower when compared to the expression induced by the chondrogenic medium, effects elicited by additives, MWCNTs, and HA + MWCNTs, in the expression of *COL6A1*, *RUNX2,* and *POU5F1* might point to low-efficient or time-shifted differentiation.

## Summary

The effects of hyaluronic acid and carbon nanotubes were investigated in 2D and 3D in vitro cell cultures. Results were concentration-dependent and differ in models (2D or 3D). HA stimulates cell viability in monolayer culture. In bioprinted constructs, MWCNTs have a beneficial influence on cell viability while HA inclusion in examined concentration has a negative impact on constructs integrity. The profile of the analyzed gene changed significantly and we observed the overall loss of transcriptional activity in most of them. These results suggest the need for more complex gene expression analysis combined with protein accumulation studies, also in extended time points. In general, promising results from 3D bioprinted scaffolds encourage undertaking in vivo tests to investigate the precise mechanism of CNTs’ interaction with cells. This may elucidate further whether they act as collagen mimetics.

## Supplementary Information

Supplementary Information.

## Data Availability

All data generated or analyzed during this study are included in this published article [and its supplementary information files].
