# Supplementary material for: Hyaluronic acid and multiwalled carbon nanotubes as bioink additives for cartilage tissue engineering
Source: Sci Rep. 2023 Jan 12;13:646. doi: 10.1038/s41598-023-27901-z (PMC9837169; doi:10.1038/s41598-023-27901-z)
Supplement: Supplementary file 1 — Supplementary Information. [file 41598_2023_27901_MOESM1_ESM.docx]

**Supplementary data**

**Supplementary Figure 1. SEM and EDX analysis.** SEM images of scaffolds without addition of the MWCNTs (A, B, C) and scaffolds supplemented with CNTs (D, E, F). EDX spectra of CNT supplemented (G) and non-supplmented scaffolds (H). There is no significant difference in atomic composition.


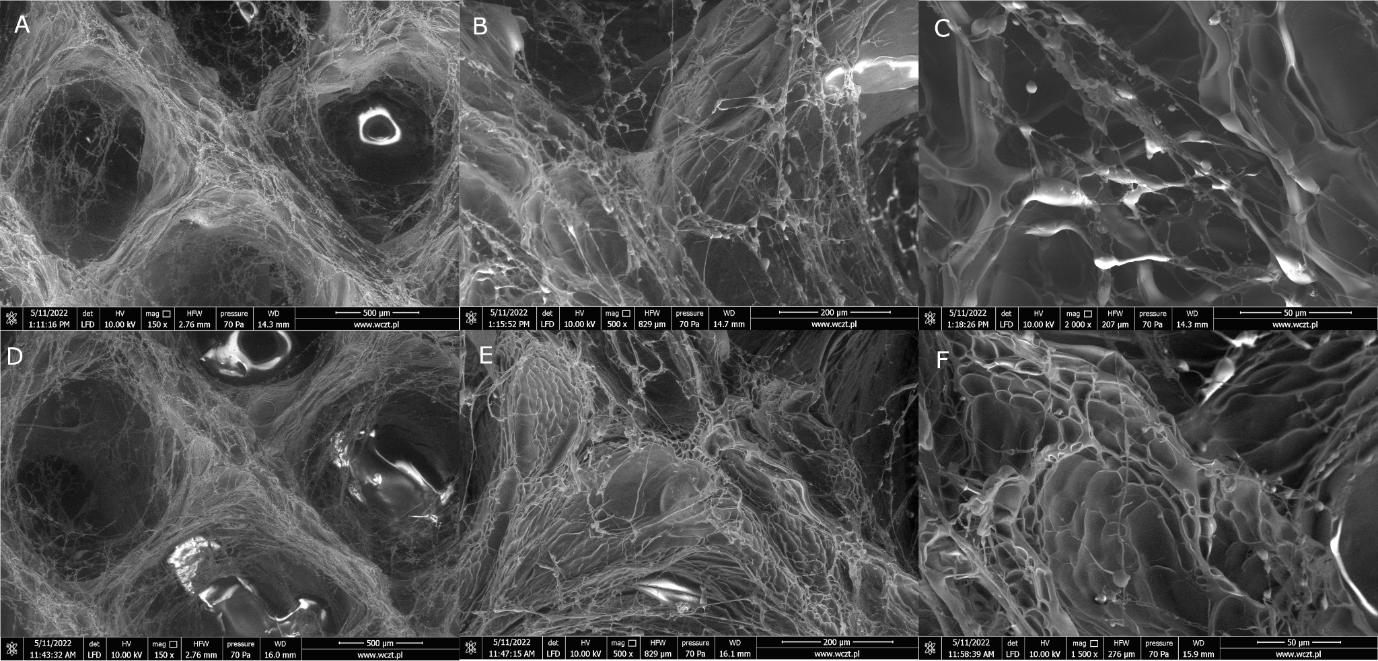

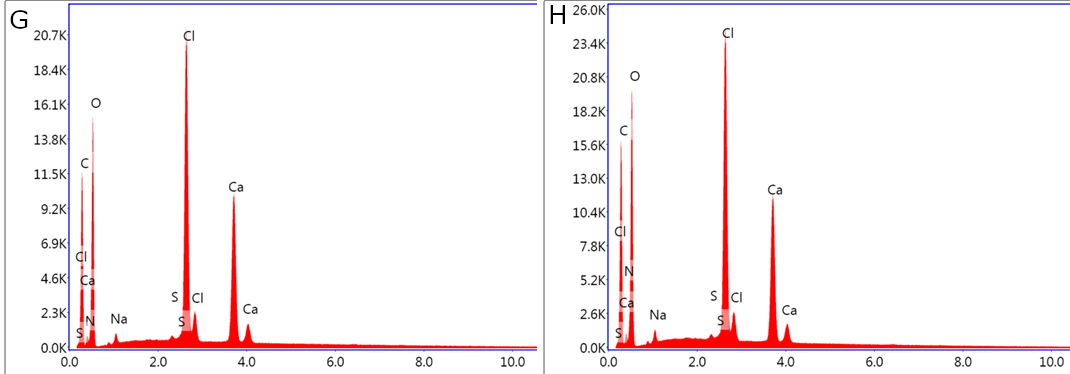


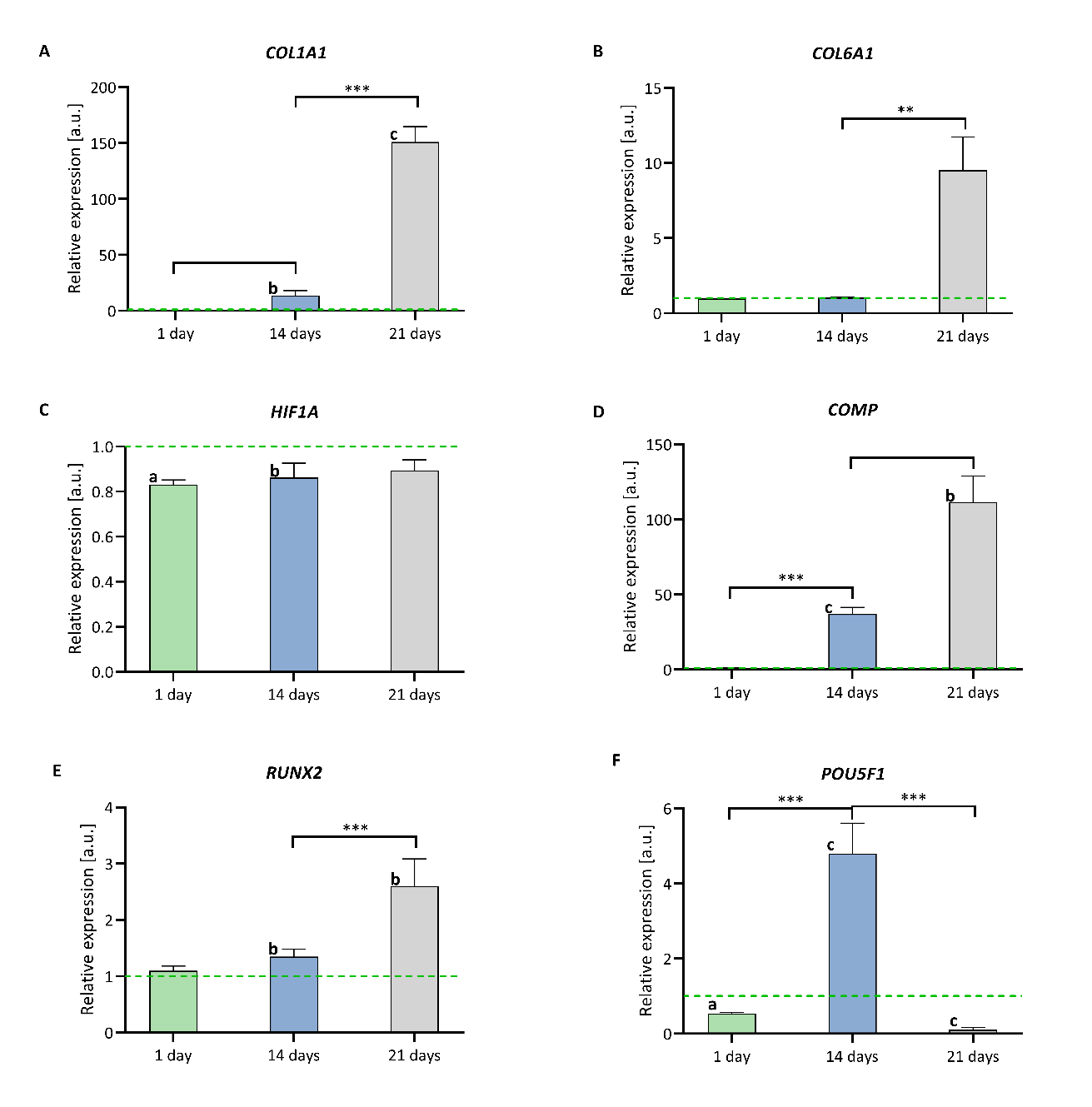


**Supplementary Figure 2**. **Differentiation medium affects expression of chondrogenic markers.** Real-time analysis of COL1A1 (A), COL6A1 (B), HIF1A (C), COMP (D), RUNX2 (E) and POU5F1 (F) gene expression in hMSCs 3D-printed with bioink without HA or MWCNTs supplementation 1 day (1d), 14 days (14d) and 21 days (21d) post-printing. Expression is normalized to average expression in hMSCs 3D-printed with bioink without additives and cultured in regular medium at particular time point (green dashed line). The statistical significance was determined by two-tailed Student’s t-test (n≥2; chondrogenic vs control: P^a^< 0.05; P^b^ < 0.01 and P^c^ < 0.001; timepoint vs time point P* < 0.05; P** < 0.01 and P*** < 0.001).

**Supplementary Tabel 1.** **Primer sequences used in real-time analysis.**

| **RPS29** | forward 5’-AGATGGGTCACCAGCAGCTGTACTG-3’ |
| --- | --- |
|  | reverse 5’-AGACACGACAAGAGCGAGAA-3’ |
| **COL1A1** | forward 5’-ACGTCCTGGTGAAGTTGGTC-3’ |
|  | reverse 5’-AGCCTCTCTCTCCTCTCTGACC-3’ |
| **COL6A1** | forward 5’-CTCGTGGACAAAGTCAAGTCCT-3’ |
|  | reverse 5’-GTAGGTGCCCTTCCCAAAGTA-3’ |
| **COL10A1** | forward 5’-TTACGCTGAACGATACCAAATG-3’ |
|  | reverse 5’-GACTTCCGTAGCCTGGTTTTC-3’ |
| **RUNX2** | forward 5’-ACCAGATGGGACTGTGGTTACT-3’ |
|  | reverse 5’-TGTGAAGACGGTTATGGTCAAG-3’ |
| **HIF1A** | forward 5’-CCAACAGTAACCAACCTCAGTG-3’ |
|  | reverse 5’-GCCTAAAAGTTCTTCTGGCTCA-3’ |
| **COMP** | forward 5’-ACAATGACGGAGTCCCTGAC-3’ |
|  | reverse 5’-TCTGCATCAAAGTCGTCCTG-3’ |
| **SOX9** | forward 5’-GACTCGCCACACTCCTCCT-3’ |
|  | reverse 5’-AGGTCTCGATGTTGGAGATGAC-3’ |
| **POU5F1** | forward 5’-GGAGATATGCAAAGCAGAAACC-3’ |
|  | reverse 5’-CTCAAAATCCTCTCGTTGTGC-3’ |
